# Incorporating environmental costs of ecosystem service loss in political decision making: A synthesis of monetary values for Germany

**DOI:** 10.1371/journal.pone.0211419

**Published:** 2019-02-13

**Authors:** Johannes Förster, Stefan Schmidt, Bartosz Bartkowski, Nele Lienhoop, Christian Albert, Heidi Wittmer

**Affiliations:** 1 Department Environmental Politics, UFZ—Helmholtz Centre for Environmental Research, Leipzig, Germany; 2 Department Computational Landscape Ecology, UFZ—Helmholtz Centre for Environmental Research, Leipzig, Germany; 3 Institute of Environmental Planning, Leibniz University Hannover, Hannover, Germany; 4 Department Economics, UFZ—Helmholtz Centre for Environmental Research, Leipzig, Germany; 5 Department Economics, Bochum University of Applied Sciences, Bochum, Germany; US Army Engineer Research and Development Center, UNITED STATES

## Abstract

Germany faces on-going degradation and biodiversity loss. As a consequence, goods and services provided by biodiversity for human well-being, so-called ecosystem services, are being lost. The associated economic costs and benefits are often unknown. To fill this gap, we conducted a literature review and developed a database of monetary values for the changes in ecosystem services that result from ecosystem change in Germany. In total, 109 monetary valuation studies of regulating and cultural ecosystem services were identified, with the majority focusing on forests and wetlands. In collaboration with valuation experts and the German Federal Environment Agency—*Umweltbundesamt* (UBA), we defined a set of criteria that economic valuation studies should meet in order to qualify for being used in decision making on national policies. Only 6 out of 109 valuation studies (5.5%) fulfilled the quality criteria for informing such decisions. Overall, monetary information on regulating and cultural ecosystem services is scattered and scarce compared to information on provisioning services, which is accounted for in detail in national statistics. This imbalance in information likely contributes to the distortion in land-use policies, giving preference to maximizing provisioning services in agricultural production and forestry, while neglecting the societal relevance of regulating and cultural services. Decision makers have to rely on only a few cost estimates that are scientifically robust, while being pragmatic to include also vague estimates in cases where data is lacking. The transferability of the monetary values included in our database depends on the biophysical and socio-economic site conditions as well as the decision context of the intended application. Case specific adjustments following guidance for benefit transfer are recommended. Given the lack of applicable studies, we call for more decision-relevant **economic** assessments. Even in cases where monetary estimates are available, we suggest decision makers to consider also other benefit information available to capture the multiple values ecosystems provide to humans.

## Introduction

Since the 19^th^ century, there is a continuous trend of ecosystem degradation in Germany that occurs unabated until today [1]. The related loss of ecosystems and their services has negative consequences for society. For example, the conversion of wetlands to agricultural land increases benefits from crop production, while causing a decline in water quality [2], the emission of soil carbon [3] and damages from flood events [4]. Key drivers of ecosystem degradation are urbanisation, land sealing for infrastructure and settlements, and conversion of grassland to cropland [1,5]. A number of national policies aim at reducing the loss of biodiversity and ecosystems, including the federal law on nature conservation (Bundesnaturschutzgesetz*)* [6], the national strategy on biological diversity [7] and the national strategy for sustainable development [8], which includes Germany’s commitment for achieving the Sustainable Development Goals (SDGs). Despite these policies and strategies, the degradation and loss of biodiversity and ecosystems continues and current changes in laws and regulations, for example, for reducing the impacts of development of urban areas, fall short in reducing land sealing and ecosystem loss [9].

Making costs and benefits of changes in ecosystem services visible through monetary values is regarded to be an effective way of informing decision making on more sustainable land-use options [10]. Ecosystems and their configuration across the landscape provide multiple ecosystem services, so-called ecosystem service bundles, for a diverse range of beneficiaries in society [11]. Current land-use decisions often focus only on a few selected ecosystem services, prioritizing provisioning services with market value (e.g. agricultural crop production) over regulating services (e.g. water provision) and cultural services (e.g. landscape aesthetics) that are typically not valued in markets [10]. Hence, land-use decisions often aim at increasing private benefits from market goods, for example from crop and timber production, while neglecting public costs and benefits related to ecosystem services, such as water regulation, carbon sequestration and landscape aesthetics. Costs related to the loss of regulating and cultural ecosystem services are mainly borne by the public, e.g. in the form of increased costs for the provision of drinking water or by damages to health [12] and occur in the form of damage costs, abatement costs or costs for replacing ecosystems with alternative man-made structures and services. Compensating or reversing the degradation and loss of biodiversity and ecosystem services through habitat restoration or replacement with man-made infrastructure and services can be expensive or is simply impossible.

As decision making is increasingly based on economic considerations, including cost-benefit analysis, there is concern that decision making will continue to ignore the costs of biodiversity and ecosystem loss [13]. Hence there is an increasing focus on including monetary values of ecosystem services in the assessment of land-use decisions in order to better account for the costs and benefits related to impacts on ecosystems and their ecosystem services [14,15]. Estimates for economic costs and benefits of land-use options can inform decision making on the multiple benefits biodiversity and ecosystems provide to human well-being as well as on the economic consequences of ecosystem loss [13,16]. For example, it has been shown that the economic benefits of conserving biodiversity and ecosystems outweigh the costs of conservation when multiple benefits of ecosystem services are accounted for [17]. Such estimates are based only on a few ecosystem services including agricultural crops, carbon sequestration, water quality, recreation (e.g. number of visitors) or willingness to pay for conservation [10,17]. The benefit-cost ratio of conserving ecosystems would increase further, if more ecosystem services were to be included in the accounting. Furthermore, while costs of protecting nature have to be considered in policy impact assessments, the inclusion of benefits from nature conservation in form of ecosystem services is optional with benefits of sustainable ecosystem management and conservation being ignored.

In Germany, the Federal Environment Agency—*Umweltbundesamt* (UBA) is aiming at establishing standardized estimates for the monetary value of ecosystem services in order to better account for changes in ecosystem services in decision making. Already today, the UBA methodological convention (UBA Methodenkonvention) provides standardized cost estimates for a range of environmental impacts. For example, the cost of carbon emission is currently estimated at 80€/tCO_2_ based on estimates of damage costs resulting from climate change [18]. This standardized cost estimate informs decision making in public procurement or regulatory impact assessment (Gesetzesfolgenabschätzung). The UBA methodological convention is currently updated with the aim of including standardized estimates for the monetary value of ecosystem services in order to inform policy processes at national level (e.g. regulatory impact assessments) on the costs of ecosystem loss.

This study provides a review of ecosystem service valuation studies available for Germany and derives recommendations for using monetary values for the changes in ecosystem services that result from ecosystem change in policy processes at national level. Challenges of monetary valuation and their implication for informing decision making are highlighted.

## Materials and methods

**Fig 1:**
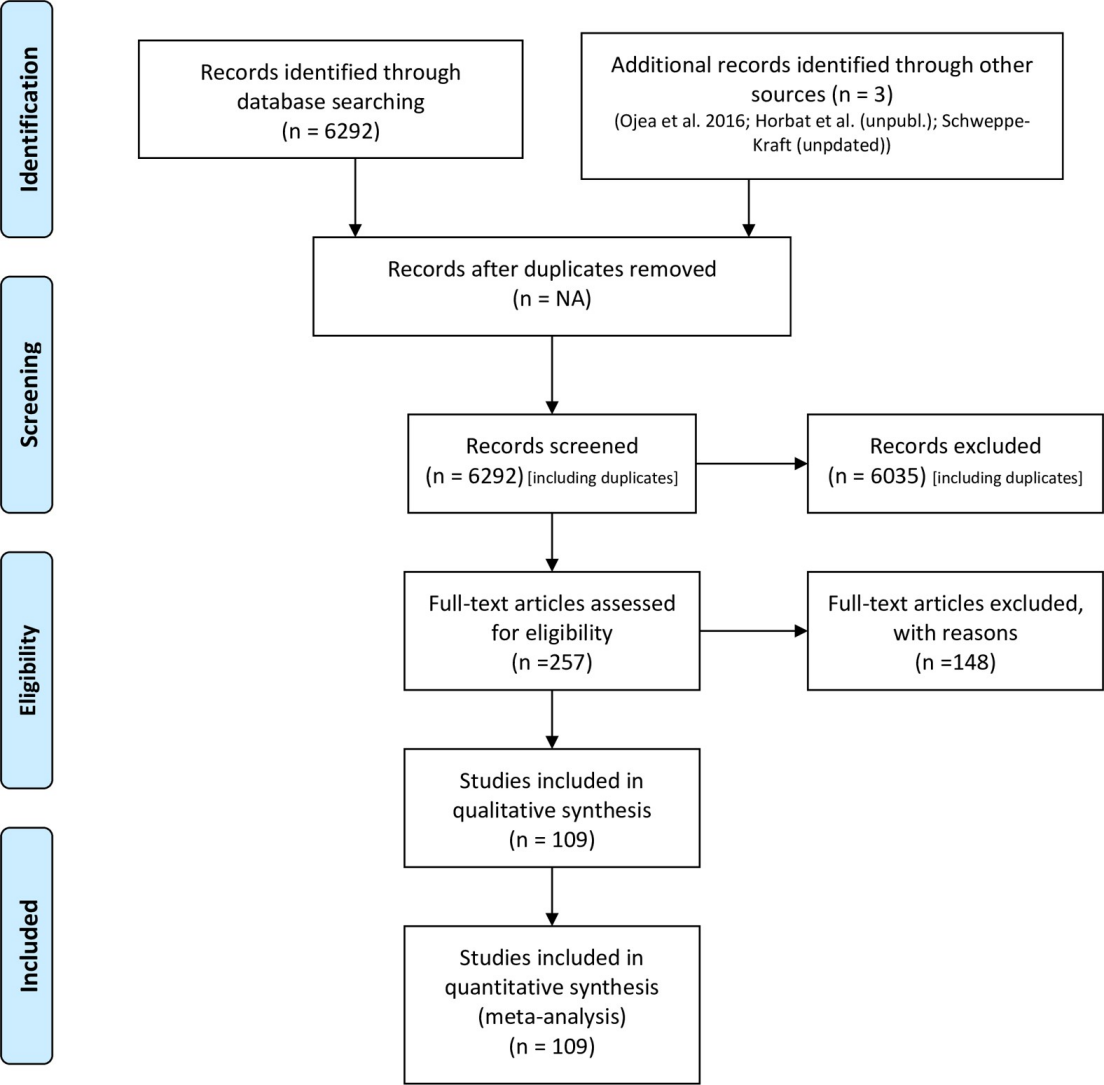
PRISMA flowchart for the identification and selection of ecosystem service valuation studies for Germany.

First, information needs were identified for updating the UBA methodological convention. Second, a literature review was conducted for developing a database with economic values for regulating and cultural ecosystem services (Fig 1, S1 Table). Third, a consultation process was conducted involving experts in ecosystem service assessment and valuation and experts from the UBA for a) ensuring quality and completeness of the literature review and b) agreeing on criteria for selecting valuation studies relevant for informing national policies. Based on the outcome of this review process, challenges and opportunities for using economic values for the changes in ecosystem services that result from ecosystem change in decision making are highlighted and recommendations for their application are derived.

### Defining information needs

A lack of information on cost estimates for ecosystem service loss has been identified for three major land conversion processes in Germany:

- Conversion of extensively or intensively used grassland into arable land (including loss of fringes of water bodies and small forest formations and coppice);
- Conversion of grassland, arable land, forests and accompanying vegetation to sealed surfaces including settlements and roads;
- Drainage of wetlands;

Given Germany’s large imports of agricultural commodities from tropical forest regions and related conversion of tropical forests with impacts on ecosystem services [19–21] tropical forest conversion was also included in the assessment:

IV Conversion of tropical rainforest into grassland or arable land.

### Literature review

For identifying studies with monetary valuation of ecosystem services related to the ecosystems and conversion processes I.—IV., bibliographic databases (e.g. Web of Science) and databases with monetary values for ecosystem services (e.g. ESVD) were searched (Table 1).

**Table 1:** Databases accessed for identification of ecosystem service valuation studies for Germany (last accessed 13 May 2016).

Name of database or publication	Total search results	Search terms	Source	Host agency
**Review of Externality Data (RED)**	84	Germany	URL: http://www.isis-it.net/red/	European Commission, Energy, Environment and Sustainable Development Programme of the Directorate General for Research
	
**Environmental Valuation Database (ENVALUE)**	422	Germany	URL: http://www.environment.nsw.gov.au/envalueapp/Default.asp?ordertype=MEDIUM	New South Wales Environmental Protection Authority, Department of Environment, Climate Change and Water
**Marine Ecosystem Service Partnership (MESP)**	1045	Germany	URL: http://www.marineecosystemservices.org/explore	Duke University, Nicholas Institute for Environmental Policy Solutions
**Environmental and Recreational (Non-Market) Values—Valuation Studies Search from National Ocean Economics Program (NOEP)**	420	Germany	URL: http://www.oceaneconomics.org/nonmarket/NMsearch2.asp	U.S. National Oceanic and Atmospheric Administration
**Ecosystem Service Valuation Database (ESVD)**	267	Germany	URL: http://es-partnership.org/services/data-knowledge-sharing/ecosystem-service-valuation-database/	Ecosystem Service Partnership (ESP) and Foundation for Sustainable Development
**Elsasser et al. 2007**	30	Germany	Elsasser, P., Meyerhoff, J., Montagné, C., and Stenger, A.: A bibliography and database on forest benefit valuation studies from Austria, France, Germany, and Switzerland–A possible base for a concerted European approach, Journal of Forest Economics, 15, 93–107, 2009	NA
**Schellhorn 1997**	18	Germany	Schellhorn, M. Instrumente der Rechenschaft über die Inanspruchnahme der natürlichen Umwelt–Umweltrechnungslegung. 2. Auflage, Wiesbaden: Dt. Univ.-Verl., Wiesbaden: Gabler, 1997, doi: 10.1007/978-3-663-09066-3	NA
**Web of Science**	1331	valuation ecosystem Germany; economic value ecosystem Germany; economic ecosystem Germany; cost ecosystem Germany; replacement cost Germany; replacement cost ecosystem Germany; damage cost ecosystem Germany; damage cost Germany; Choice experiment ecosystem Germany; Choice experiment Germany; Willingness to pay ecosystem Germany; Willingness to pay Germany; cost ecosystem Germany; restoration cost ecosystem Germany; economic wetland Germany; economic grassland Germany; economic wetland Germany; economic agriculture Germany; economic forest Germany;	URL: http://apps.webofknowledge.com	Thomson Reuters
**Datenbank „Dokumentation Natur und Landschaft—online”(DNL-online)**	647	Bewertung Ökosystem Deutschland; Ersatzkosten Ökosystem; Schadenskosten Ökosystem; Wiederherstellungskosten Ökosystem; Choice (Experiment) Ökosystem; Zahlungsbereitschaft Ökosystem; Kosten Ökosystem; Wiederherstellung Ökosystem; Nutzen Ökosystem Deutschland; Nutzen Ökosystem	URL: http://www.dnl-online.de	Bundesamtes für Naturschutz (Bonn, Leipzig, Insel Vilm).
**Karlsruhe Virtueller Katalog (KVK)**	1977	Bewertung Ökosystem Deutschland; ökonomische Bewertung Ökosystem; Ersatzkosten Ökosystem; Schadenskosten Ökosystem; Wiederherstellungskosten Ökosystem; Wiederherstellungskosten; Choice (Experiment) Ökosystem; Zahlungsbereitschaft Ökosystem; Kosten Ökosystem; Wiederherstellung Ökosystem; Nutzen Ökosystem Deutschland; Bewertung Ökosystem; Bewertung Flächen; Wert Flächen; Bewertung Grasland; Bewertung Grünland; Wert Grasland; Wert Grünland; Wert Ackerland; Bewertung Ackerland;	URL: http://kvk.bibliothek.kit.edu/	KIT-Bibliothek Karlsruher Institut für Technologie (KIT)
**Ojea et al. 2016**	51	-	Ojea et al. (2016) Ecosystem Services and REDD: Estimating the Benefits of Non-Carbon Services in Worldwide Forests. *World Development (78) 246–261*.	NA

Both peer-reviewed and grey literature were considered. Although grey literature is often not peer-reviewed, it can be a useful complementary resource [22]. Provisioning services such as agricultural production and timber production are not considered in this review, as these ecosystem services are already captured in land-use statistics at local and national level [23]. Instead, the focus of this review is primarily on regulating and cultural services that are usually not captured in land-use statistics. The review focuses on primary valuation studies for ensuring complete recording of information on biophysical and socio-economic context, study design, valuation methods, and underlying assumptions (S1 Database contains a full list of all recorded variables and values). Given that the outcome of monetary valuation studies is highly dependent on the context of the study area and the choice of valuation method, the following characteristics were recorded in the database:

- full reference of study
- ecosystem service classified according to The Economics of Ecosystems and Biodiversity (TEEB) [24] and Common International Classification of Ecosystem Services (CICES) [25]
- spatial and temporal dimension (area of study site, location, year of valuation etc.)
- information on biophysical and socio-economic context
- valuation method, sample size and underlying assumptions
- discount rate
- monetary value (minimum, mean, median, maximum)

Various valuation methods exits for assessing the economic costs of ecosystem service loss associated with land-use changes [26,27]. A fundamental element of the ecosystem service paradigm is the recognition that changes in ecosystems influence the provision of ecosystem services, and that these changes in services have influence on human welfare. In economic terms, an increase in the flow of ecosystem services is regarded as benefits and a decrease in flows is regarded as costs. These benefits and costs reflect the preferences of individual stakeholders affected by the change. Both market and non-market valuation methods can be used to estimate the change of economic value associated with the changes in ecosystem services flow. Market valuation means economic values are derived from market prices. Examples include the forgone economic value of agricultural products or timber, which is sold on a market (market analysis) due to expansion of settlements, the costs of offset activities to compensate for a new road (restoration costs) or increased water treatment costs due to soil erosion when grassland is converted to arable land (damage cost). Many ecosystem services are not traded in markets and therefore have no market price. In this case, it is necessary to assess the economic value of a decreased flow of ecosystem services through direct or indirect non-market valuation methods. Direct methods (also called stated preference methods) refer to contingent valuation (CV) and choice experiments (CE), where the affected general public is asked directly in a survey for their willingness to pay (WTP) to obtain a land-use change (to value the benefits of an increased ecosystem services flow) or their WTP to avoid a land-use change (to value the costs of a decreased ecosystem services flow). WTP can also be obtained indirectly by assuming that economic value is reflected in the costs incurred by travelling to specific sites, such as recreational visits to wetland areas (travel cost method), or additional property prices paid to live in specific environment, e.g. in the vicinity of a forest (hedonic pricing method). In the latter two approaches, economic value is ‘revealed’ through observable behaviour [26,27]. These approaches apply also for assessing the willingness to accept (WTA) possible negative consequences.

### Reporting of monetary values in database

Monetary values derived from the reviewed literature are recorded in a database. Monetary values were inflation-adjusted to 2014 values using the consumer price index (CPI) [28] (Eq 1). Estimates in Deutsche Mark (DM) were converted to Euro (€) using the general currency conversion factor of 1 Euro = 1.95583 DM.

$${Value\;in€}_{2014}={Value}_{reported}*\frac{{CPI}_{2014}}{{CPI}_{Year\;of\;valuation}}$$ (1)

Values in other currencies were inflation-adjusted using the consumer price index of the respective country (Eq 1). For allowing comparability of values across countries, values were adjusted for purchasing power parity (PPP) [29] (Eq 2).

$${Value\;in€}_{2014}={Value}_{2014}*\frac{{PPP}_{Germany}}{{PPP}_{Country\;of\;valuation\;study}}$$ (2)

In a third step, values were converted to groups of similar units:

- €/ha/a
- €/ha
- €/Person/a
- other

The classification used for ecosystem services follows CICES of the European Environmental Agency. In order to ensure compatibility with existing databases, similar categories for valuation methods were used as in the database by de Groot et al. (2012) [30]. When converting values of larger study areas into values per hectare, linear scale effects were assumed, dividing the aggregate monetary value by the number of hectares. Values per household were divided by the average number of household members in Germany (1.99 members) based on the German Federal Statistical Office [31]. For studies from other countries, household values were divided by the respective average number of household members (S1 Database). Similarly, inflation-adjustment and currency conversions are based on assumptions of data homogeneity.

### Identification of monetary values

During a two-day workshop, the results of the literature review were assessed by external experts on ecosystem service assessments and monetary valuation for ensuring quality and completeness of the literature review. This ensures that the most relevant valuation studies for biodiversity and ecosystem services are included in the database. The workshop was designed as a means for presenting and discussing preliminary findings with distinguished experts. The participants were thus not part of the generation of research results, but rather reflected upon the results' comprehensiveness and validity. In consequence, no approval from an ethics committee was required.

In order for monetary values to be representative for the conversion processes I.—IV. and to inform decision making at national level, the monetary values obtained from the literature review must allow for generalization. In consultation with experts from the UBA, criteria a.–g. were identified for evaluating the suitability of valuation studies for deriving cost estimates for ecosystem service loss:

- The thematic focus of the valuation study is at least on one of the relevant conversion processes and ecosystems (I.–IV.);
- There is an explicit description of biophysical and socio-economic context;
- Transparency of study design, methods and underlying assumptions;
- Monetary values refer to a distinct, clearly identifiable ecosystem service or ecosystem service bundle;
- Monetary values are derived using common valuation methods (cost-based or benefit-based approaches);
- Monetary values are reported in Euro per hectare (ii. €/ha) or allow for currency conversion and unit-adjustment;
- Representativeness of monetary values: the reasoning for minimum–maximum ranges of values is explained by a minimum–maximum range in biophysical or socio-economic factors (e.g. carbon content of ecosystem per hectare).

## Results

**Fig 2:**
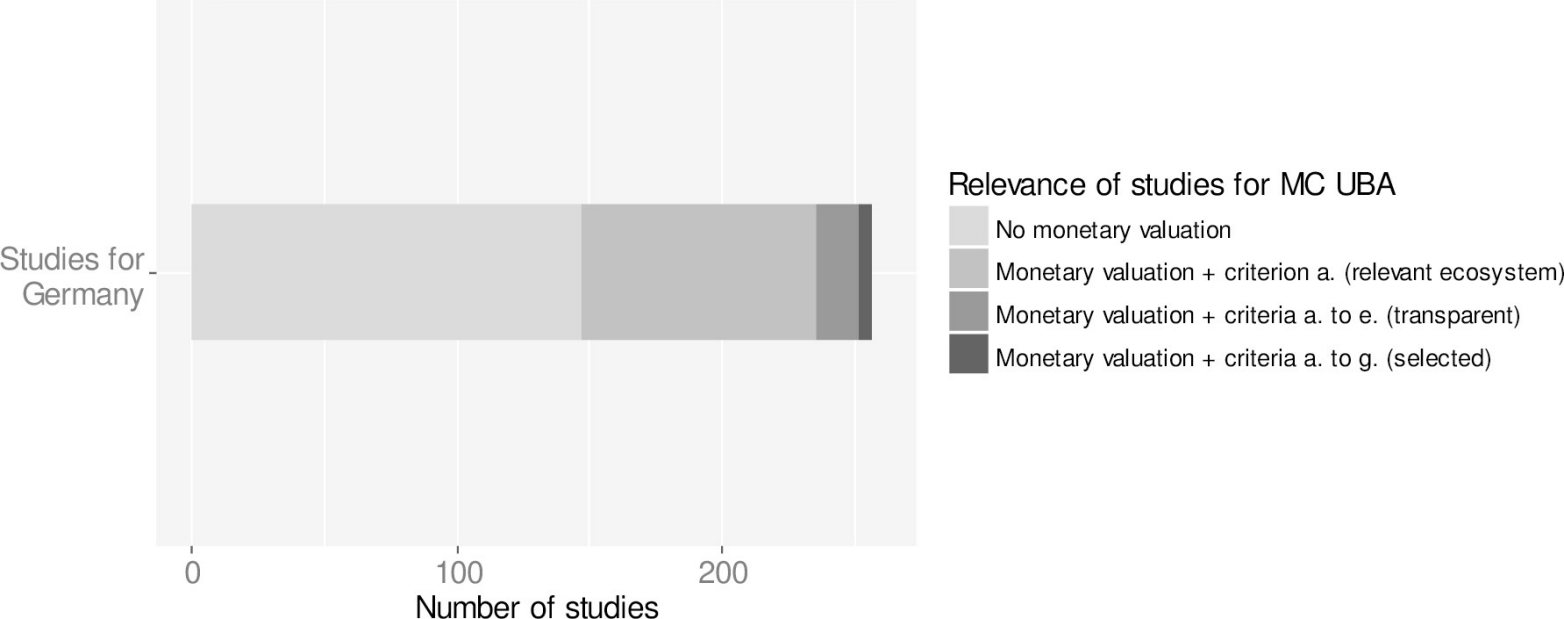
Number of monetary valuation studies for changes in ecosystem services in Germany. In total, 257 studies with a focus on ecosystem service valuation in Germany were reviewed. 148 studies do not estimate monetary values or only cite values from other valuation studies. 109 studies are primary valuation studies, estimating monetary values for ecosystem services related to conversion processes I-III (including grasslands, arable lands, wetlands, forests and sealed surfaces). Of the 109 studies 21 studies are sufficiently transparent (complying with selection criteria a. to e.). Of the 21 studies only six studies comply with all selection criteria (a. to g.) by being sufficiently transparent, reporting monetary values in a common unit (e.g. € per ha) and minimum-maximum ranges can be explained by minimum-maximum ranges in biophysical or socio-economic context. Only these studies were selected for informing the methodological convention (MC) of the Umweltbundesamt (UBA).

**Fig 3:**
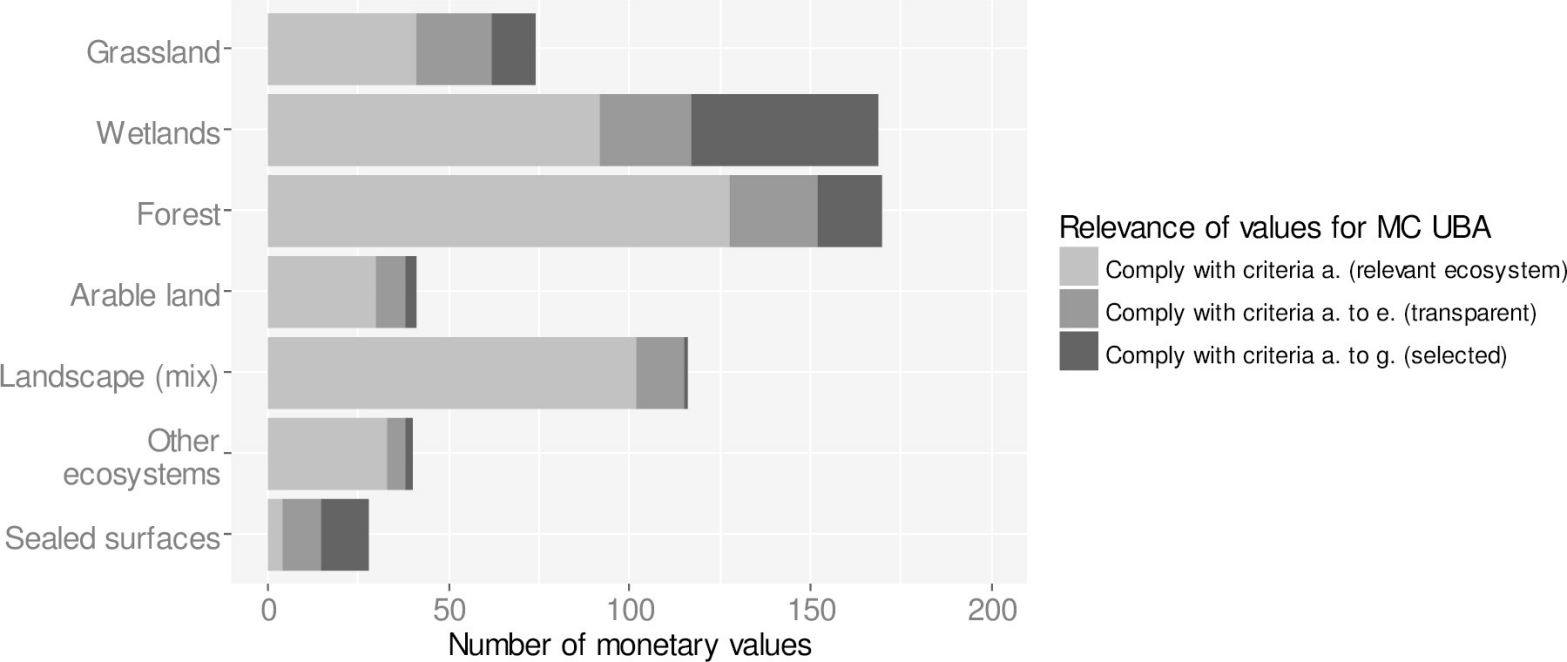
Number of monetary values for changes in ecosystem services of common land-cover types in Germany. The database contains 633 monetary values for ecosystem services from 109 primary valuation studies that focus on at least one of the ecosystems involved in the conversion processes (I.—IV.). The majority of monetary values have been identified for forests and wetlands. 15 studies with 204 monetary values are sufficiently transparent and comply with selection criteria a. to e. Six studies with 101 monetary values comply with all selection criteria (a.—g.) and have highest relevance for informing the methodological convention (MC) of the Umweltbundesamt (UBA) on possible costs involved in the loss or degradation of ecosystem services.

Based on the review of literature and existing databases, 257 studies were identified with a thematic focus on ecosystem service valuation in Germany and a focus on ecosystems and land cover types related to conversion processes I.—III. (grasslands, arable lands, wetlands, forests and sealed surfaces) (Fig 2; S1 Database). Of the 257 studies 109 turned out to be distinct valuation studies with a total of 638 monetary values for changes in ecosystem services (Fig 3). The largest number of monetary values is available for wetlands (n = 169) and forests (n = 170). 21 out of 109 studies comply with the selection criteria a. to e. with study design and information on biophysical and socio-economic context being sufficiently transparent. Only six studies comply with all selection criteria a. to g. providing 101 monetary values. These studies were used for informing the UBA methodological convention on possible costs involved in the loss or degradation of ecosystem services (Table 2).

**Table 2:** Studies with monetary values of changes in ecosystem services complying with criteria for informing national policies (criteria are defined in methods).

Reference	Publication type	Focus of valuation	Ecosystem service (CICES code)	Related conversion process	Minimum ranges of monetary values (inflation-adjusted with 2014 as base year)	Comment
Born et al. 2012 [33]	Project report	Benefit of wetlands for nutrient retention. Replacement costs for alternative approaches for removing nitrate N.	Water quality (retention of nitrate N and phosphate P) (2.3.4.)	III. Wetland conversion.		
				Benefit of wetland for removing 1 kg N:	6.16€/kg N	
				Benefit of one hectare wetland for N retention	663.27–809.07 €/ha	
Born et al. 2012 [33]	Project report	Benefit of wetlands for nutrient retention. Replacement costs for alternative approaches for removing phosphate P.	Water quality (retention of nitrate N and phosphate P) (2.3.4.)	III. Wetland conversion.		
				Benefit of wetland for removing 1 kg P:	61.60€/kg P	
				Benefit of one hectare wetland for P retention:	159.14€/ha	
Grossmann 2012 [34]	Peer-reviewed publication	Benefit of each additional hectare inundated/ restored wetland for N and P retention.	Water quality (retention of nitrate N and phosphate P) (2.3.4.)	III. Wetland conversion. Restoring riparian wetland area for achieving reduction in N and P load by:	Benefit of one additional hectare of inundated/restored wetland for N and P retention:	
				Scenario 1 (S1): 5%	S1: 1,636–1,834 €/ha	
				Scenario 2 (S2): 15%	S2: 1,2665–1,3059 €/ha	
				Scenario 3 (S3): 25%	S3: 21,173–25,028 €/ha	
				Scenario 4 (S4): 35%	S4: 43,189–56,557 €/ha	
Horbat et al. (2016), [35] S1 Database	Project report	Valuation of benefit of wetland restoration for N retention.	Water quality (retention of nitrate N) (2.3.4.)	III. Wetland conversion.		Peer-reviewed publication of data is recommended.
				Scenario 1 (S1): Restoring riparian wetland area from 4748 ha to 6426 ha:	S1: 649.51 €/ha/year	
				Scenario 2 (S2): Restoring riparian wetland area from 4748 ha to 8494 ha	S2: 233.22 €/ha/year	
Horbat et al. (2016), [35] S1 Database	Project report	Valuation of benefit of wetland restoration for P retention.	Water quality (retention of phosphate P) (2.3.4.)	III. Wetland conversion.		Peer-reviewed publication of data is recommended
				Scenario 1: Restoring riparian wetland area from 4748 ha to 6426 ha.	S1: 615.72 €/ha/year	
				Scenario 2: Restoring riparian wetland area from 4748 ha to 8494 ha.	S2: 229.11 €/ha/year	
Ott et al. (2006) [36]	Project report	Cost of habitat restoration	Biodiversity (habitat, species) (2.3.1.)	II. Restoration of sealed surfaces.	9,273.91–9,4265.21 €/ha (net present value)	Requires update of underlying assumptions.
Reutter and Matzdorf (2013) [37]	Book chapter	Monetary valuation of nitrate (N) retention and leakage to freshwater as result of changes in intensity of grassland use.	Water quality (retention of nitrate N) (2.3.4.)	I. Grassland conversion.		Underlying assumptions of monetary values for N and P retention could be updated using 6 € per kg N and 60 € per kg P.
				Scenario 1: low intense use of grassland to high intense use of grassland (increase of N emissions: 20 kg N/ha/year)	S1: 10.92 €/ha/year	
				Scenario 2: low intense use of grassland to arable land (increase of N emissions: 70 kg N/ha/year)	S2: 65.63 €/ha/year	
Schweppe-Kraft (update based on Schweppe-Kraft 1998) [38,39]	Report from 1998 updated in 2016 (unpublished update)	Cost of habitat restoration: grasslands	Biodiversity (habitat, species) (2.3.1.)	I. Grassland conversion. Restoration of grasslands of different habitat quality.	31,811.17–91,457.11 €/ha (net present value)	Based on habitat-valuation-point system. Monetary value per habitat-point is based on Schweppe-Kraft (1998). Requires update.
Schweppe-Kraft (update based on Schweppe-Kraft 1998) [38,39]	Report from 1998 updated in 2016 (unpublished update)	Cost of habitat restoration: forests	Biodiversity (habitat, species) (2.3.1.)	II. Forest restoration. Restoration of forests of different habitat quality.	43,740.35–91,457.11 €/ha (net present value)	Requires update (see above).
Schweppe-Kraft (update based on Schweppe-Kraft 1998) [38,39]	Report from 1998 updated in 2016 (unpublished update)	Cost of habitat restoration: wetlands	Biodiversity (habitat, species) (2.3.1.)	III. Wetland conversion. Restoration of wetlands of different habitat quality.	67,598.73–95,433.50 €/ha (net present value)	Requires update (see above).
Ojea et al. (2016) [32]	Peer-reviewed publication	Benefit from tropical forests	Physical experience (recreation) (3.1.1.)	IV. Tropical forest (no conversion).	682.91 €/ha/a	Based on meta-analysis of multiple valuation studies
Ojea et al. (2016) [32]	Peer-reviewed publication	Benefit from tropical forests	Biodiversity (habitat, species) (2.3.1.)	IV. Tropical forest (no conversion).	3960.74 €/ha/a	Based on meta-analysis of multiple valuation studies
Ojea et al. (2016) [32]	Peer-reviewed publication	Benefit from tropical forests	Ecosystem service bundle: air quality and water regulation (excluding carbon)	IV. Tropical forest (no conversion).	5287.27 €/ha/a	Based on meta-analysis of multiple valuation studies
Ojea et al. (2016) [32]	Peer-reviewed publication	Benefit from tropical forests	Ecosystem service bundle: "food and fibre"	IV. Tropical forest (no conversion).	4267.11 €/ha/a	Based on meta-analysis of multiple valuation studies

**Fig 4:**
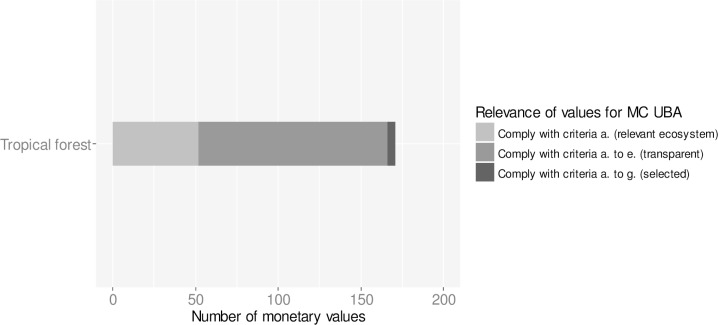
Number of monetary values for changes in ecosystem services of tropical forests. Tropical forests are addressed in conversion process IV. The database contains 171 monetary values for ecosystem services of tropical forests from a total of 23 monetary valuation studies. Of the 171 monetary values 114 comply with criteria a. to e. with regards to the transparency of study design and methods. Five aggregated monetary values from the review by Ojea et al. (2016) **[32]** comply with criteria a.—g. and have highest relevance for informing the methodological convention (MC) of the Umweltbundesamt (UBA) on possible costs involved in the loss or degradation of ecosystem services.

Monetary values for changes in ecosystem services provision in tropical forests were derived from already existing databases including de Groot et al. (2012) [30] and the literature review by Ojea et al. (2016) [32] (Fig 4). From the 23 studies with 171 monetary values, 114 values comply with the criteria on transparency (criteria a. to e.). Five aggregated monetary values based on the meta-analysis of Ojea et al. (2016) [32] comply with criteria a. to g and have been selected for informing the UBA methodological convention.

**Fig 5:**
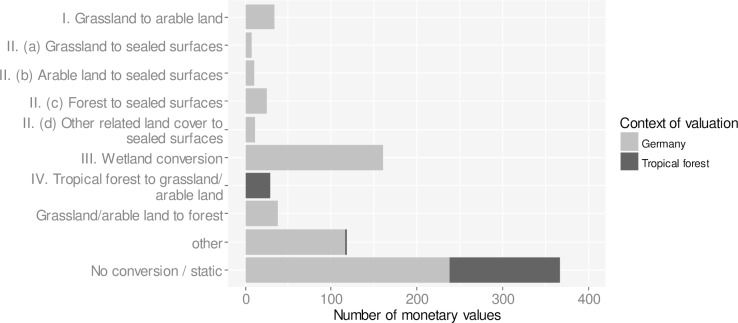
Number of monetary values for ecosystem services impacted by ecosystem conversion processes. Almost half (46%, n = 375) of the monetary values for ecosystem services originate from valuation studies that estimate stocks or marginal changes within the same ecosystem type (no conversion). 36% of monetary values (n = 288) address one of the four relevant conversion process (I.—IV.). Wetland conversion (III.) is the process with most monetary values (n = 161).

In total, the database contains 809 monetary values from 132 valuation studies for ecosystems in Germany and tropical forests. Almost half of the monetary values (46%, n = 375) provide estimates for stocks or marginal changes in ecosystem services within the same ecosystem type (Fig 5). About one third of monetary values (36%, n = 288) address ecosystem conversion processes (I.—IV.). Wetland conversion is the process for which most monetary values of ecosystem services are available (20%, n = 161), including estimates for wetland restoration.

**Fig 6:**
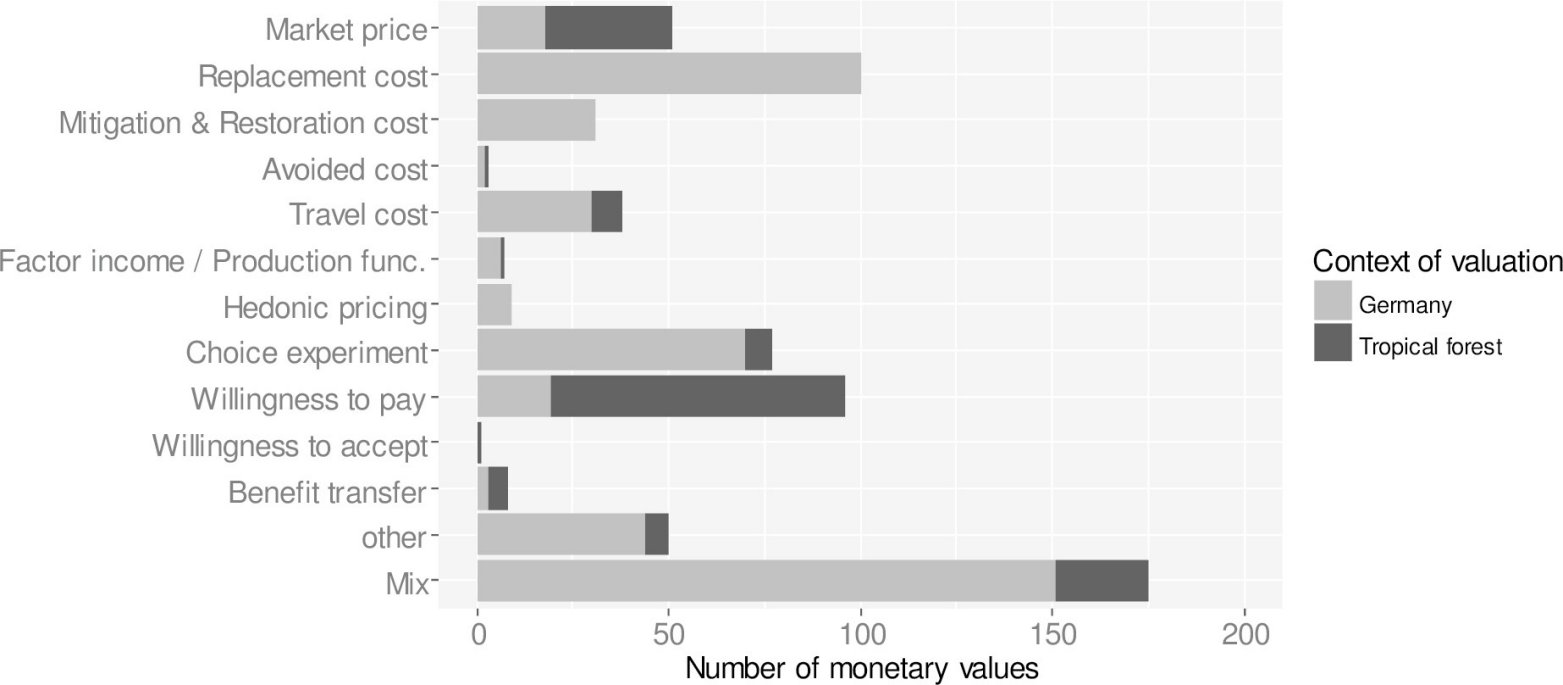
Monetary valuation methods. The majority of monetary values originate from valuation studies that apply a mix of valuation methods. Using replacement costs as means for valuing ecosystem services is a common approach in Germany, followed by choice experiments. In tropical regions, willingness to pay and market price methods dominate.

The monetary values originate from studies with a diversity of valuation methods. In total, about 11 major groups of valuation methods have been identified (Fig 6). Using replacement costs as means for valuing ecosystem services is the most common approach in Germany, followed by choice experiments. For valuing ecosystem services of tropical forests, willingness to pay and market price methods dominate. However, the majority of monetary values originate from valuation studies that apply a mix of valuation methods.

**Fig 7:**
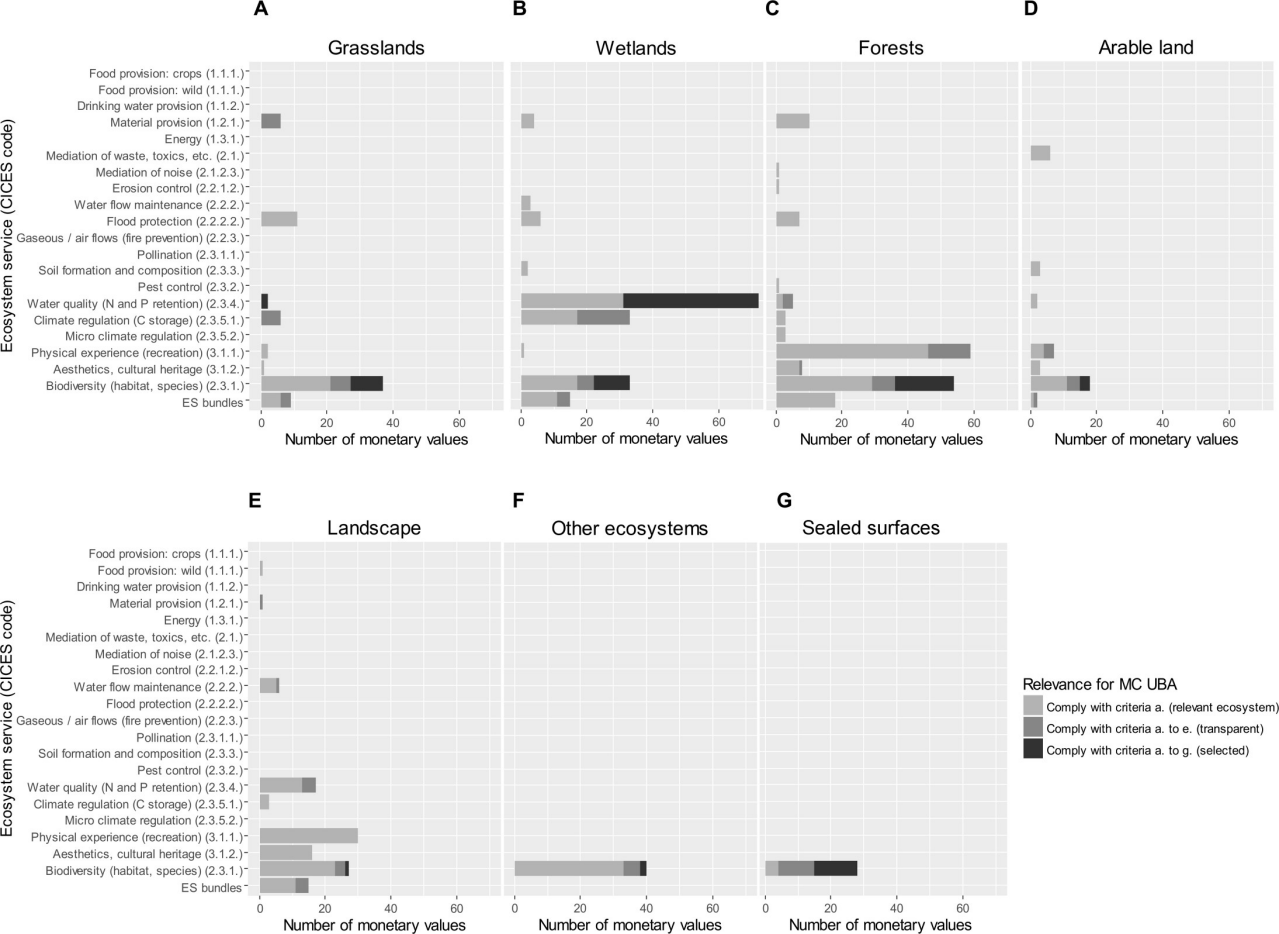
**A–G. Number of monetary values for changes in ecosystem services in Germany (ecosystem services classified according to CICES).** The panels show the number of monetary values for changes in ecosystem services available for A grasslands, B wetlands, C forests, D arable land, E landscapes, F other ecosystems, and G sealed surfaces. The panels include all ecosystem services classes addressed by the 109 reviewed valuation studies for Germany. The class “Biodiversity (habitat, species) (CICES 2.3.1.)” is valued most frequently across all ecosystem types (A to G) and includes the appreciation of people for ecosystems to provide habitat for species and diversity of ecosystems across landscapes. Ecosystem services with a high number of monetary values also include “Physical experience (recreation) (CICES 3.1.1.)” in particular for forests (panel C) and “Water quality (N and P retention) (CICES 2.3.4.)” in particular for wetlands (panel B). **Note:** Agricultural production and timber production are not considered in this review as these ecosystem services are already captured in land-use statistics at local and national level [23].

**Fig 8:**
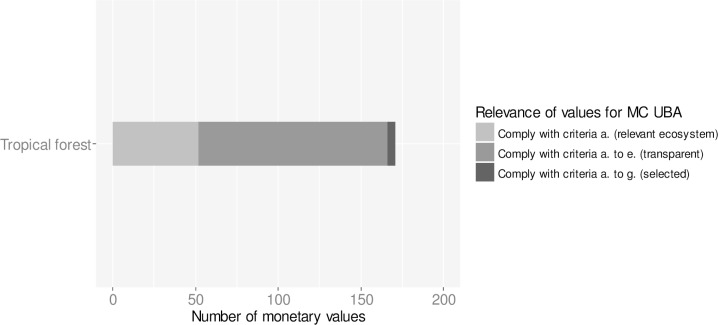
Number of monetary values for changes in ecosystem services in tropical forests (classified according to CICES). The class “Biodiversity (habitat, species) (CICES 2.3.1.)” is valued most frequently and includes the appreciation of people for ecosystems to provide habitat for species and diversity of ecosystems across landscapes. This is followed by the distinct ecosystem service classes “Food provision: wild (CICES 1.1.1.)”, “Material provision (CICES 1.2.1.)” and “Physical experience (recreation) (CICES 3.1.1.)”.”Ecosystem service bundles” with multiple ecosystem service classes are also frequently valued. Colour coding indicates the relevance of values for informing the methodological convention (MC) of the Umweltbundesamt (UBA) on possible costs involved in the loss or degradation of ecosystem services.

According to the CICES, the reviewed valuation studies address 20 ecosystem service classes (Fig 7A–7G and Fig 8). Some of the studies also value bundles of ecosystem services, e.g. the joint valuation of recreation, aesthetics and habitat provision for biodiversity.

For ecosystems in Germany, the class “Biodiversity (habitat, species) (CICES 2.3.1.)” is valued most frequently (n = 237). It includes the appreciation of people for ecosystems to provide habitat for species and related diversity within ecosystems and across landscapes. Ecosystem services with a high number of monetary values also include “Physical experience (recreation) (CICES 3.1.1.)” in particular for forests (n = 50) and “Water quality (N and P retention) (CICES 2.3.4.)” in particular for wetlands (n = 72). Agricultural production and timber production are not considered, as the focus of this assessment is on regulation and cultural ecosystem services.

For tropical forests, frequently valued ecosystem services include “Biodiversity (habitat, species) (CICES 2.3.1.)”, “Food provision (CICES 1.1.1.), bundles of multiple ecosystem services, “Material provision (CICES 1.2.1.)” and “Physical experience (recreation) (CICES 3.1.1.)” (Fig 8).

### Selected ecosystem service valuation studies

Based on the selection criteria (a. to g.), six ecosystem service valuation studies for Germany and one meta-analysis of valuation studies for tropical forests were selected for informing the UBA methodological convention on potential costs in terms of ecosystem service loss (Table 2).

As example of how a standard cost estimate for a change in an ecosystem service can be used to inform on economic costs involved in land-cover conversions, the standard cost estimate of 80€/tCO_2_, which is currently used by the UBA for estimating damage costs of carbon emissions, is applied to the carbon balance of land-cover change reported by the German Government under the Kyoto Protocol (S2 Table; [40]).

## Discussion

In Germany, regulatory impact assessments evaluating new policy proposals increasingly rely on monetary cost-benefit analysis, which often do not consider the costs of ecosystem service loss. This study provides a first systematic and comprehensive review of monetary valuation studies of changes in ecosystem services for common ecosystems and land-cover conversion processes in Germany. In addition, this review includes information on the potential costs of ecosystem service loss caused by tropical deforestation, which is relevant for accounting for the costs of ecosystem service loss due to imports of agriculture and forest commodities from tropical forest regions [19–21]. As such, this literature review and the developed database can inform on potential costs and benefits involved in land-cover change in terms of ecosystem services loss in Germany and tropical forest regions.

Gaps in knowledge on the economic dimension of the benefits biodiversity and ecosystems contribute to human well-being in Germany were identified. Considering that provisioning services, including agricultural production and forestry, are accounted for in detail in local and national statistics [23], the identified 109 studies with monetary values for changes in regulating and cultural ecosystem services in Germany since the 1980s are strikingly small in number. This confirms concerns that current decision making processes are distorted, giving preference to maximizing provisioning services in agricultural production and forestry, while neglecting the relevance of regulating and cultural services for society [10].

Furthermore, only 6 out of 109 ecosystem service valuation studies (5.5%) were found to comply with all selection criteria (a. to g.) for informing policy impact assessments targeted by the UBA methodological convention (Fig 7 and Table 2). This highlights the need for monetary valuation studies to be more policy relevant by: i) being more transparent and robust with regards to study design and valuation methods, by ii) assessing and reporting information on ecosystem services in common and comparable units (e.g. providing information on biophysical and socio-economic indicators in values per hectare and/or per capita) and, if possible, by iii) explaining minimum-maximum ranges in monetary values by measurable changes in biophysical or socio-economic indicators (Fig 7). This would enhance the interpretation of the reported monetary values on changes in ecosystem services in light of the original valuation studies and allow for judging their suitability, credibility, and reliability for informing regulatory impact assessment and decisions on policy design.

The majority of valuation studies focus only on a few ecosystems and ecosystem services (Fig 7), revealing blind spots in the literature on ecosystem service valuation. Forests and wetlands have received greatest attention in ecosystem service valuation in Germany (Fig 3) with a focus on habitat provision for biodiversity, recreation, and nutrient (N and P) retention for freshwater quality (Fig 7). Other regulating and cultural ecosystem services are less frequently assessed, including pollination, soil formation, erosion control and pest control. Potential explanations for the focus of valuation studies on forests and wetlands include that there is a long history of research on these ecosystems in Germany. Biodiversity, recreation and water quality are also topics of public interest and therefore such research is more likely to be supported by donors and decision makers, while other ecosystem services are less visible and neglected in scientific assessments.

One of the identified gaps includes the lack of literature on monetary valuation of ecosystem services of grasslands. While grasslands are heavily affected by land-cover conversion in Germany [1,5], only 14 studies were found to address the monetary value of ecosystem services of grasslands (Fig 3). Reutter and Matzdorf 2013 [37] estimate that the intensification of grassland use increases nitrate (N) emissions by 20 kg per hectare and year, while the conversion of grasslands to arable land increases nitrate emissions by 70 kg per hectare and year, causing monetary costs of about 10.92–65.63 €_2014_ per ha per year (Table 2). These are costs society is bearing as a result of grassland loss.

The use of fertilizers on agricultural land and the lack of natural ecosystems buffering nitrate from reaching freshwater systems is a major cause for the continuous increase in nitrate concentrations. As consequence, it is expected that the resulting increase in efforts for purifying drinking water from nitrate will increase the costs for water users by 32 to 45%[41,42]. Currently, the European Commission has taken legal steps against the German government due to continuously high nitrate concentrations in water bodies in Germany, which exceed the thresholds of the European Union Nitrate Directive [43]. Due to the lack of effective measures and policies for reducing nitrate concentrations, the German government is facing the payment of significant fines.

In the following, challenges and limitations of using monetary values in decision making are discussed for the identified studies.

Monetary values for nutrient retention allow for generic conclusions on the benefits ecosystems provide in terms of capturing nutrients (nitrate N and phosphate P) (e.g.[33–35], Table 2). Currently, standard estimates for the replacement cost of nutrient retention are at 6.16 € per kg N and 60.60 €_2014_ per kg P (e.g. [33], Table 2). However, it is important to note that these estimates are only a partial reflection of the true costs that incur to society when nutrients enter freshwater systems. These cost estimates are based on the replacement cost method assuming costs that would incur, if nutrient loads in the water were to be reduced through technical measures for water treatment. However, the replacement costs do not include damage costs caused by excess nutrient loads in freshwater systems causing species loss, impacts on human health, deterioration of water quality and related decline in aesthetic and recreational values.

Given these shortcomings of the replacement cost method, it is recommended to use the damage cost method for estimating the costs of ecosystem service loss. This recommendation is in line with already existing guidance by the UBA methodological convention. For example, the cost of carbon emissions is based on estimates for damages caused by climate change impacts, which are currently estimated to be about 80 €/tCO_2_ [18]. This estimate for the social cost of carbon emissions has become an established reference in Germany. It is used, for example, for determining the cost of wetland degradation and the benefits of restoring wetlands for mitigating carbon emissions [44]. Applying this cost estimate to the biophysical carbon values used in the national reporting under the Kyoto Protocol [40] allows for a rough estimation of the costs caused by carbon emissions from land-cover change in Germany (S2 Table). Using similar standardized biophysical indicators and monetary estimates (e.g. for nutrient retention N and P) could allow for a better recognition of ecosystems benefits in decision making at national scale.

For some of the selected studies an update of the monetary estimates is recommended, using more recent information on the monetary benefits of biodiversity and ecosystem services. For example, Schweppe-Kraft (1998) [38] provides restoration costs for a diversity of habitats based on the habitat-valuation-point system (Biotopwertpunkte). This habitat-valuation-point system is used for assessing the ecological quality of habitats and is well-established in land-use planning in Germany. It is applied, for example, in environmental impact assessments (EIA) for informing decision making on options for conserving, mitigating, restoring and offsetting environmental impacts. Although the monetary values presented by Schweppe-Kraft (Table 2) take into account a recent update of the habitat-valuation-point system, the underlying economic model used for determining the monetary value of a single habitat-valuation point is based on an outdated value of the willingness to pay for biodiversity conservation from the 1990s (Schweppe-Kraft 1998). As the socio-economic context of the 1990s, when the original study was conducted, is very different from today’s context (e.g. due to changes in income, unemployment rates, demography, etc.), the use of monetary values of past valuation studies within today’s reality involves large uncertainties [45]. Therefore, an update of the monetary values of Schweppe-Kraft (Table 2) is recommended, using more recent estimates for the monetary benefits of biodiversity and ecosystem services. The same applies for studies following similar methodological approaches, such as Ott et al. (2006) [36].

For ecosystem services of tropical forests, the study by Ojea et al. (2016) [32] provides monetary values based on a review of existing valuation studies and a meta-analysis using linear regression analysis. While such an analysis can provide an important contribution to establishing more general estimates for the monetary value of ecosystem services, one has to be aware that the context of the original valuation studies is lost in the process of the meta-analysis. The original valuation studies have been designed for addressing a particular research or policy question within a specific biophysical and socio-economic context and at a specific spatial scale (e.g. local or national). This information is not contained in the aggregated values. Therefore, it is critical to consult the original valuation studies when interpreting and using aggregated values for informing decision making.

As shown with the examples above, one has to be aware that monetary estimates for changes in ecosystem services are only “snapshots” of a few selected costs or benefits and economic values of ecosystem services account only for a subset of benefits biodiversity and ecosystem services provide to human well-being [46]. In addition, monetary valuation of non-market goods–a characteristic that applies to most regulating and cultural ecosystem services–involves methodological and conceptual challenges. In particular the loss of multiple values, including cultural and intrinsic values, is not being represented in monetary estimates [46]. Each ecosystem provides bundles of multiple ecosystem services with a great diversity of values and benefits [47]. However, ecosystem service assessments often focus on a few selected ecosystem services, neglecting the benefits of multiple ecosystem services. Valuation methods can also address only certain aspects of benefits, with multiple values not being accountable in monetary terms. Hence, monetary values of changes in single ecosystem services should be interpreted as minimum values and the total cost of ecosystem loss is likely to be larger.

Given the shortcomings discussed above, relying exclusively on monetary values is a narrow approach to decision making. It can lead to outcomes in favour of or against biodiversity conservation [48]. Hence, economic valuation should be regarded to be an illustration of potential economic costs involved in decision options that should be complemented also by other methods and indicators that allow for integration of multiple values of biodiversity and ecosystem services in decision making [46]. Furthermore, decision makers do not want to rely only on economic information and demand also other types of information [49] and taking into account the multiple values of biodiversity and ecosystems, including their intrinsic and cultural values, is essential for an inclusive decision making process [50].

### Recommendations on the use of monetary values for changes in ecosystem services

The monetary values recorded in the database and the selected values presented in Table 2 can provide a first indication on the benefits regulating and cultural ecosystem services provide to human well-being in Germany. However, it is important to be aware of the methodological challenges of monetary valuation of ecosystem services in order to judge the credibility and suitability of monetary values for informing decision making. Monetary values for ecosystem services should not pretend to communicate absolute values but explicitly denote relative changes in the value of ecosystem services that result from biophysical and socio-economic changes related to ecosystem changes. Monetary valuation is done on a case-by-case basis [51] and therefore the judgment of the credibility and suitability of monetary values for informing decision making should also follow a case-by-case approach. The monetary value of a change in an ecosystem service depends on the particular biophysical and socio-economic site conditions [52]. Often, monetary values cannot be directly transferred and applied to other sites without careful adjustment to the particular local conditions of the target site. Best-practice guidance on the use of benefit transfer (e.g. Johnston et al. 2015 [53] should be followed when using the monetary values of this database. Due to the diversity in the biophysical and socio-economic context of study sites and the diversity in valuation methods (Fig 6), the suitability of monetary values for benefit transfer requires a thorough review of the primary valuation studies. When using benefit transfer, it should be also demonstrated and justified why a monetary value fits the context and purpose of the particular decision making context. If an original monetary valuation study insufficiently describes the biophysical and socio-economic conditions of the study site and lacks clarity in the used methods, then we recommend to not use the monetary values for benefit transfer. Furthermore, as ecosystem service valuation studies use a great diversity of methods (Fig 6), it is not advised to aggregate monetary values across different valuation studies. Instead, ranges of minimum and maximum values should be used for ecosystem services in order to reflect the diversity in valuation methods and socio-ecological contexts.

Our findings highlight that decision making has to account for the trade-off in relying on few cost estimates for ecosystem services that are scientifically robust, while being pragmatic enough to include also vague estimates from studies that may not comply with the defined selection criteria (a.–g.). This review and the generated database can serve as tool for identifying ecosystem service valuation studies that are relevant for informing decision making processes. However, the database should not be used as a one-stop-shop for an arbitrary use of monetary values. The database can guide the identification of monetary valuation studies of ecosystem services relevant for informing decision making, but it does not replace a careful assessment of the original valuation studies for informing a particular decision context.

Finally, it has been shown for spatial planning that decision makers prefer a mix of multiple indicators that allow weighing decision options for different criteria within a specific decision context [54,55]. Multi-dimensional frameworks as, for example, multi-criteria decision analysis (MCDA), allow for the inclusion of quantitative and qualitative information on multiple values of biodiversity and ecosystems. This can open up discourses in decision making processes and help reflect the views and values of multiple stakeholders [12,46,56–58]. Therefore, the use of monetary values of ecosystem services should be accompanied also by information on other indicators in order to allow for inclusive decision making processes that take into account the multiple values of biodiversity and ecosystem services.

## Conclusions

This review highlights significant gaps in knowledge on the monetary value for the changes in ecosystem services that result from ecosystem change in Germany and a lack of studies relevant for informing decision making. Therefore, it is recommended that future ecosystem service valuation studies should better target the specific information needs of decision makers in order to provide information on ecosystem service indicators that are relevant for informing decision making at local and national level. While using monetary values on ecosystem services can open up the debate on the relevance of biodiversity and ecosystem services for society and inform the design of policies including cost-benefit analysis, decision making should not only rely on single monetary values for ecosystem services. This would bear the risk of underestimating the benefits of ecosystem services and costs involved in ecosystem loss. Given that biodiversity and ecosystem services provide multiple values [59], it should be recognized that monetary valuation is only one approach of many for assessing the importance of nature for human well-being [46].

## Supporting information

S1 Table Reporting of systematic review. (DOCX)

S2 Table Carbon balance of major ecosystem conversion processes in Germany and related ecosystem service costs and benefits. (XLSX)

S1 Database Monetary values of changes in ecosystem services in Germany. (XLSX)
